# Emulsifier-Modulated Microstructure of Soy Protein–Arabinoxylan Oleogels Improves Astaxanthin Bioaccessibility and In Vivo Antioxidant Activity

**DOI:** 10.3390/foods15081315

**Published:** 2026-04-10

**Authors:** Xiaolong Shen, Wenhao Hu, Wenrong Meng, Tiancheng Sheng, Xiuhong Zhao, Jiaxin Li, Qingyu Yang, Longkun Wu

**Affiliations:** School of Food Science and Technology, Shenyang Normal University, Shenyang 110031, China

**Keywords:** soy protein, arabinoxylan, emulsifier, astaxanthin, oleogel

## Abstract

Astaxanthin (AST), despite its high bioactivity, exhibits poor stability and low bioavailability due to its strong lipophilicity and inherent degradation susceptibility. To overcome such a challenge, we developed a food-grade oleogel delivery system using a soy protein–arabinoxylan (SA) glycosylated complex modulated by different concentrations (0.5–3%) of sucrose ester (SE) or soy lecithin. We show that the emulsifier concentration has a non-linear effect on the oleogel microstructure: an optimal level of 1% had a significant impact on the interfacial compactness and network density, giving rise to improved thermal stability, rheological strength and AST encapsulation efficiency (81.27%). During in vitro digestion, the SA matrix in combination with emulsifiers allowed gastric protection and intestinal-targeted release of AST with a bioaccessibility of up to 88.84% (SAO-SE-AST). This controlled release profile directly translated into enhanced in vivo antioxidant efficacy in wild-type Bristol N2 *Caenorhabditis elegans*, as evidenced by reduced lipofuscin accumulation, elevated thermotolerance (survival rate: 64.44–73.33%), suppressed reactive oxygen species levels and activation of endogenous antioxidant enzymes (superoxide dismutase as well as glutathione peroxidase). Collectively, this research has uncovered that food-grade emulsifiers are not only stabilizers, but also key regulators of oleogel architecture and bioactive functionality. These results provide a structure–digestion–bioactivity correlation for protein–polysaccharide oleogels, representing a rational design strategy for high-performance delivery systems of lipid-soluble nutraceuticals.

## 1. Introduction

Astaxanthin (AST) is a powerful natural carotenoid with outstanding antioxidant activity, far exceeding the activity of β-carotene and vitamin E—because of its distinctive molecular structure with conjugated double bonds as well as terminal hydroxyl/keto groups [1,2]. Beyond its free radical scavenging capacity, AST exhibits complex bioactivities, like anti-inflammatory, neuroprotective as well as anti-atherosclerotic effects, making it a promising ingredient for functional foods and nutraceuticals [3,4,5]. However, the extreme lipophilicity, chemical instability in the presence of light and oxygen, and poor gastrointestinal bioavailability limit the AST practical application [1,6]. These limitations require the development of sophisticated delivery systems which can increase the stability of AST during processing and allow for controlled release in the gut to maximize absorption.

Encapsulation technology is an effective strategy for protecting bioactivity. In this context, oleogels, which are structured liquid oils encapsulated in a 3D gel network, have become a promising platform for the delivery of lipid-soluble bioactives [7,8,9]. In contrast to traditional emulsions or solid lipid nanoparticles, oleogels provide better physical stability, adjustable rheological properties, and the possibility to control the release of nutrients based on the matrix design [10,11,12]. Recent studies further indicated the choice of structuring agent critically influence bioaccessibility; for instance, glyceryl monostearate-based oleogels significantly enhanced AST bioavailability compared with wax-based systems [11]. Nevertheless, most existing oleogel formulations are based on synthetic or nonfood-grade gelators, which limits their use in clean-label food products.

Protein–polysaccharide complexes are a sustainable alternative, with synergistic effects of protein–polysaccharide complexes to create strong interfacial films and gel networks [13,14]. Maillard-derived soy protein–arabinoxylan (SA) conjugate combines the emulsifying capacity of soy protein and the thickening and stabilizing properties of the arabinoxylan, so as to produce a biocompatible matrix with enhanced thermal alongside oxidative resistance [15,16]. While SA complexes hold promise for encapsulation, their behavior in oleogel systems, especially the effect of food-grade emulsifiers on modulating microstructure, digestion behavior and in vivo bioactivity, is poorly understood. Although sucrose ester (SE) and soy lecithin (SL) are widely used to enhance interfacial stability, the concentration-dependent effects of these emulsifiers on SA-based oleogel formation, AST retention, and biological functionality have not been systematically elucidated.

This study aimed to construct AST-loaded oleogels using the SA glycosylated complex as a food-grade gelator, and systematically investigate the modulatory effects of SE and SL at varying concentrations (0.5–3%) on the physicochemical structure, in vitro gastrointestinal digestion behavior, and in vivo antioxidant efficacy of the delivery system. While protein–polysaccharide oleogels and emulsifier-modified systems have been reported individually, the systematic investigation of how emulsifier concentration non-linearly modulates the SA-based oleogel architecture and its consequential impact on the complete trajectory from in vitro digestion to in vivo antioxidant efficacy remains elusive. The *Caenorhabditis elegans* (*C. elegans*) model was selected for in vivo validation herein, owing to its highly conserved antioxidant signaling pathways with mammals, short lifespan, and well-established protocols for assessing oxidative stress and thermotolerance, which make it a physiologically relevant model to predict the in vivo performance of nutraceutical delivery systems in higher organisms. We hypothesized that emulsifier-mediated optimization of the oleogel 3D network would enhance AST encapsulation stability, gastrointestinal bioaccessibility, and subsequent in vivo antioxidant activity. By integrating material characterization, simulated gastrointestinal digestion, and *C. elegans* bioassays, we sought to establish a quantitative structure–function relationship linking emulsifier-mediated network design to biological outcomes. This study fills this gap by establishing a robust structure–digestion–bioactivity correlation, demonstrating that food-grade emulsifiers serve as critical architectural regulators beyond their conventional stabilizing role, thereby providing a rational basis for engineering high-performance, label-friendly delivery systems for sensitive lipophilic nutraceuticals.

## 2. Materials and Methods

### 2.1. Materials

Soy protein isolate (SPI, purity > 90 g/100 g) was purchased from Henan Zhongchen Biotechnology Co., Ltd. (Zhengzhou, China). Arabinoxylan (AX) (Xi’an Rongzhen Biotechnology Co., Ltd., Xi’an, China), Sucrose ester (SE, S-1170, HLB value = 11; esterification degree: 70%; monoester content: ~50%) and soy lecithin (SL, HLB value = 7) were purchased from Jiahe Xuri Food Co., Ltd. (Chenzhou, China). *Acer truncatum* seed oil was purchased from Jiangxi Yisenyuan Plant Spice Co., Ltd. (Ji’an, China). Wild-type *C. elegans* Bristol N2 strain and uracil-deficient *Escherichia coli* OP50 strain were purchased from Hangzhou Hongsai Biotechnology Co., Ltd. (Hangzhou, China). All nematodes used in experiments were age-synchronized hermaphrodites at the L4 larval stage, with no prior experimental procedures or genetic modification. Superoxide dismutase (SOD), malondialdehyde (MDA), and glutathione peroxidase (GSH-Px) kits (Beijing Solarbio Science and Technology Co., Ltd., Beijing, China) were also leveraged. Octane, ammonium thiocyanate (ATC), cumene hydroperoxide, trichloroacetic acid, 2-thiobarbituric acid, 1,1,3,3-tetramethoxypropane, ferrous sulfate, barium chloride, as well as tert-butanol were all of analytical grade and purchased from Shandong Keyuan Biochemical Co., Ltd. (Yantai, China).

### 2.2. Preparation of the Oleogel Precursor Emulsion and Oleogel

SPI and AX were conjugated through Maillard glycosylation, with all reaction conditions selected based on prior work [15]. The two materials were mixed at a pre-validated 2:1 mass ratio, dissolved in deionized water to obtain a 6 wt.% aqueous solution, and adjusted to pH 9.0 using sodium hydroxide. The glycosylation reaction was performed under the conditions described in the literature [17]. The solution was first incubated in a 60 °C water bath, followed by seven incubation cycles in an 83 °C water bath to ensure full glycosylation. The successful glycosylation of SPI and AX (Degree of Glycosylation: 21.46%) was previously confirmed in our established protocol [15], validating the covalent conjugation prior to oleogel formation. Existing studies on protein–polysaccharide composite oleogel systems have shown that the effective working concentrations of food emulsifiers in such systems mostly fall within the 0.1–3% range. Concentrations below 0.1% cannot achieve effective modification of the oil–water interface, while those above 3% tend to reduce the stability of the emulsion system due to competitive interfacial adsorption and self-aggregation of emulsifier molecules [18,19]. To determine the appropriate addition level of emulsifiers for oleogel fabrication, a pre-experimental screening was first conducted over a wide concentration range of 0.1% to 5% (calculated based on the mass of the oil phase), with oil binding capacity (OBC) as the core performance indicator for the structural stability of oleogels (Supplementary Figure S1). Pre-experimental results revealed a non-linear concentration-dependent effect of SE and SL on the OBC of oleogels: the emulsifier-mediated enhancement of gel network stability was extremely weak at concentrations below 0.5%, reached the maximum at 1%, and showed a cliff-like decline when the concentration exceeded 3% due to interfacial supersaturation and molecular overcrowding. On this basis, three representative mass concentrations of 0.5%, 1% and 3% were selected for subsequent formal experiments, which corresponded to the sub-optimal regulation level, the optimal structural modulation point, and the supramolecular crowding threshold of emulsifiers in the SAO system, respectively.

Briefly, SE or SL at the above set concentrations was added to Acer truncatum seed oil (0.1 wt.% AST), and the mixture was stirred at 400 rpm for 30 min at 80 °C. The as-prepared composite aqueous solution was mixed with A. truncatum seed oil at a mass ratio of 7:3 and homogenized at 14,000 rpm for 5 min. Subsequently, emulsions including the blank soy protein isolate–arabinoxylan emulsion (SAE), SAE–0.5%SL, SAE–1%SL, SAE–3%SL, SAE–0.5%SE, SAE–1%SE, and SAE–3%SE were obtained and stored at 4 °C for later use. The final emulsions were frozen overnight at −80 °C in an ultra-low temperature freezer, and then freeze-dried under vacuum for 72 h to obtain dried products. The products were then ground at 4000 rpm for 15 min in a high-speed mill to obtain the corresponding soy protein isolate–arabinoxylan oleogels (SAO), namely blank SAO, SAO–0.5%SL, SAO–1%SL, SAO–3%SL, SAO–0.5%SE, SAO–1%SE, and SAO–3%SE (corresponding to blank SAO, SAO–0.5%SL–AST, SAO–1%SL–AST, SAO–3%SL–AST, SAO–0.5%SE–AST, SAO–1%SE–AST, and SAO–3%SE–AST, respectively).

### 2.3. Measurement of Zeta Potential and Particle Size

A Zetasizer Nano ZS 90 nanoparticle size and potential analyzer (Malvern Panalytical Ltd., Malvern, UK) was leveraged to measure the oleogel emulsion’s zeta potential and particle size. Particle refractive index 1.450, dispersant (water) refractive index 1.330, and absorbance 0.001 were the specified instrument settings. Each sample was measured three times, and all tests were conducted at 25 °C.

### 2.4. Microstructure Observation

To observe the oleogel microstructure, the sample was immersed in petroleum ether for 0.5 h to remove oil; the process was repeated three times to ensure complete removal. Subsequently, the deoiled gel skeleton was seen under an SU8020 scanning electron microscope (Hitachi High-Technologies Corporation, Tokyo, Japan) 1.0 kV.

### 2.5. Oil Binding Capacity (OBC)

The calculation of the oil loss was based on a previously reported procedure with minor adjustments [20]. In short, 1 g of oleogel was put in a 5 mL centrifuge tube as well as centrifuged at 7155× *g*/15 min. Exuded oil was dried after 30 min of standing and filter paper was leveraged to take out the oil after inversion. The loss of oil was estimated in the way:

(1) $$Oil\;loss(\%)=(1-\frac{M_{2}-M}{M_{1}-M})\times 100$$

where M_1_ denotes the total mass of the sample and centrifuge tube before centrifugation (g), M_2_ represents the mass of the sample and centrifuge tube after centrifugation and oil absorption (g), and M indicates the mass of the empty centrifuge tube (g).

### 2.6. Thermal Properties

A differential scanning calorimeter (DSC-250, TA, New Castle, DE, USA) was leveraged to analyze the oleogels’ thermal behavior [21]. Approximately 5 mg of sample was hermetically sealed in an aluminum T-zero pan with a lid (TA Instruments, New Castle, DE, USA). An empty sealed pan served as reference. The instrument was calibrated for temperature and enthalpy using indium (Tm = 156.6 °C, ΔH = 28.45 J/g) and zinc standards. Measurements were performed under a nitrogen purge flow of 50 mL/min to prevent oxidative degradation. The temperature program called for heating to 200 °C at 10 °C per minute after equilibration at 40 °C.

### 2.7. Fourier Transform Infrared Spectroscopy (FTIR)

FTIR spectra were attained on a Vertex 70 spectrometer (Bruker Company, Karlsruhe, Germany) following. Samples were directly applied to the ATR crystal and scanned from 4000 to 400 cm^−1^ at a resolution of 4 cm^−1^ over 32 accumulations [22].

### 2.8. Rheological Properties

Rheological characterization was performed using a Haake RheoStress 6000 rheometer (Thermo Fisher Scientific, Waltham, MA, USA) [23]. Using a 40 mm parallel-plate geometry with 1 mm gap, tests comprised frequency sweeps (0.1–10 Hz, 0.1% strain), strain sweeps (0.01–1%, 1 Hz, 25 °C), and temperature sweeps (5–80 °C). Thixotropic behavior was evaluated via steady-state shear tests, determining apparent viscosity (η) at shear rates of 0.1 and 10 s^−1^.

### 2.9. AST Encapsulation Efficiency Analysis

Encapsulation efficiency (EE) of AST was quantified following a modified literature procedure [24]. A total of 1 mg of oleogel was blended with 2 mL ethanol, stirred magnetically for 1 min, as well as centrifuged (4 °C, 800 rpm, 10 min) to discard unencapsulated AST. The encapsulated fraction was then extracted with 1 mL dichloromethane/methanol (2:1, *v*/*v*) as well as recovered by centrifugation (4 °C, 12,000 rpm, 10 min). The extraction was repeated three times to make sure of completeness. Absorbance of the combined extract was measured at 480 nm via a UV–Vis spectrophotometer (Lambda 365, PerkinElmer, Waltham, MA, USA), with the solvent mixture as blank. AST content was derived from a standard curve, and EE was calculated as follows:

(2) $$\mathrm{EE}(\%)\;=\;\frac{m_{0}}{m}\times 100$$

where m suggests the total mass of AST in the oleogel and m_0_ denotes the mass of the embedded AST.

### 2.10. Oxidative Stability Analysis

The oleogel’s oxidative stability was assessed by accelerated oxidation testing. Samples were placed in test tubes and stored in the dark at 40 °C for 12 days. Samples were gathered on days 0, 3, 6, 9, and 12 to determine the peroxide value (PV) as well as thiobarbituric acid active substance (TBARS) value [25]. A 0.2 g oleogel sample was incorporated to 1.5 mL of an isooctane–isopropanol mixture (3:1, *v*/*v*), vortexed three times (10 s each), and 200 μL of the supernatant organic layer was collected. Subsequently, 2.8 mL of a methanol–butanol mixture (2:1, *v*/*v*), 50 μL of ATC solution, and 50 μL of 0.072 M ferrous chloride solution were incorporated. After mixing, the sample was left for 10 min, and absorbance was measured at 510 nm. The PV was computed. The TBARS determination procedure was performed as follows: 0.15 g of oleogel sample was reacted with an aqueous solution containing 15 wt.% trichloroacetic acid, 0.375 wt.% butyric acid, as well as 2 wt.% hydrochloric acid. The mixture was heated in a 90 °C water bath for 20 min, cooled to room temperature, and centrifuged at 1370× *g* for 15 min. The supernatant was measured at 532 nm, and the TBARS concentration was computed based on the 1,1,3,3-tetraethoxypropane standard curve.

### 2.11. Antioxidant Capacity Assay

Using previously published techniques, the DPPH free radical scavenging activity was measured [26,27]. To create a working solution, a suitable quantity of DPPH was weighed, dissolved in ethanol, and diluted to a final concentration of 25 μg·mL^−1^. In short, 0.5 mL of sample solution (made with pH 7, 20 mM PBS, sample concentration 1 mg·mL−1) was mixed with 2 mL of the DPPH working solution, vortexed, and allowed to react for 60 min in the dark. After centrifuging the sample for 15 min at 10,000 rpm, the absorbance of the supernatant was measured at 517 nm (A_1_). As a control, an equivalent volume of PBS was utilized (A). Following the manufacturer’s instructions, the FRAP assay kit from Nanjing Jiancheng Bioengineering Research Institute, Nanjing, China was leveraged to measure total antioxidant capacity. The following formula was used to get the DPPH free radical scavenging rate:

(3) $$\mathrm{DPPH}\;\mathrm{removal}\;\mathrm{rate}(\%)=\frac{A\;-\;A_{1}}{A}\;\times \;100$$

where A and A_1_ represent the absorbance of the control as well as the sample.

### 2.12. In Vitro Simulated Gastrointestinal Digestion

The in vitro digestion protocol was adapted from the INFOGEST 2.0 standardized static model with minor modifications [28]. Specifically, the gastric phase duration was set to 1 h based on preliminary release kinetics showing >90% gastric emptying of the marker within this timeframe for our solid oleogel matrices (as opposed to the 2 h recommended for liquid meals). Enzyme activities (pepsin: 2000 U/mL; pancreatin: 800 U/mL trypsin activity) were verified via azocasein assay prior to each experiment.

The release of free fatty acids (FFAs) during oleogel digestion was quantified via a modified pH-stat method, as displayed in prior work [29]. Simulated gastric fluid (SGF) was formulated by incorporating 3.2 mg·mL^−1^ pepsin, 2 mg·mL^−1^ sodium chloride, and 0.7% (*v*/*v*) hydrochloric acid. For gastric digestion, 2 g of oleogel was combined with 20 mL SGF at 37 °C and stirred continuously (100 rpm, 1 h) while maintaining pH 2.5. Subsequently, the pH of the digestate was adjusted to 7.0 before blending with simulated intestinal fluid (SIF) at a 1:3 (*v*/*v*) ratio. SIF contained 5 mg·mL^−1^ bile salt extract, 150 mM sodium chloride, 1.6 mg·mL^−1^ pancreatin, as well as 10 mM calcium chloride. Intestinal digestion proceeded for 2 h under continuous stirring (100 rpm), with pH maintained at 7.0 via dropwise addition of 0.1 M NaOH.

#### 2.12.1. FFA Release Kinetics

To assess the degree of lipolysis, the FFA release rate was calculated via the following [29]. The average molar mass of Acer truncatum seed oil (MLipid = 932.9 g/mol) was calculated based on its fatty acid composition [30] (C16:0 4.29%; C18:0 2.41%; C18:1 22.94%; C18:2 33.33%; C18:3 1.90%; C20:0 0.25%; C20:1 8.01%; C22:0 0.84%; C22:1 16.61%; C24:1 5.88%).

(4) $$FFA\;release\;rate(\%)=\frac{V_{NaOH}\times C_{NaOH}\times M_{Lipid}}{2\times W_{Lipid}}\times 100$$

where V_NaOH_ denotes the volume of NaOH solution consumed (L), C_NaOH_ signifies the concentration of NaOH solution (mol·L^−1^), W_Lipid_ represents the total mass of oil in the oleogel (g), and M_Lipid_ indicates the average molar mass of *Acer truncatum* seed oil (g·mol^−1^).

#### 2.12.2. AST Release Kinetics and Bioavailability

The AST release rate as well as final bioavailability during digestion were identified built on previously reported methods [31]. Samples were gathered every 10 min from the gastric stage (SGF) or intestinal stage (SIF) after the start of digestion and centrifuged at 12,000 rpm for 10 min to obtain the supernatant. AST was gathered from the supernatant, and its content was determined based on the approach displayed in Section 2.8. AST release rate as well as bioavailability are calculated using the following formula:

(5) $$\mathrm{AST}\;\mathrm{release}\;(\%)\;=\frac{C_{1}}{C}\times 100$$

(6) $$\mathrm{Bioavailability}\;(\%)=\frac{C_{2}}{C}\times 100$$

where C represents the initial AST content in the oleogel prior to digestion, C_1_ denotes the AST content measured in the supernatant, and C_2_ signifies the total amount of AST released at the end of digestion.

### 2.13. In Vivo Antioxidant Activity Study

All in vivo experiments were conducted using a parallel group design. Age-synchronized *C. elegans* were randomly assigned to the following treatment groups: a blank control group (without AST and oleogel), an oil control group (*Acer truncatum* seed oil only), oleogel control groups (SAO, SAO-SL, and SAO-SE), an SAO-AST group (SAO without emulsifier, loaded with AST), an SAO-SL-AST group (SAO modified with 1% SL, loaded with AST), and an SAO-SE-AST group (SAO modified with 1% SE, loaded with AST). The experimental unit was a single nematode, with 30 individuals per treatment group. Each treatment group was evaluated in independent biological replicates. All experiments were performed with three independent biological replicates conducted on separate days, with freshly prepared reagents and culture medium used for each replicate.

#### 2.13.1. Cultivation and Inoculation of *E. coli* OP50

OP50 is a uracil-deficient *E. coli*, whose growth on NGM medium is limited. In this experiment, using OP50 as food for *C. elegans* avoids excessive proliferation that could affect *C. elegans* growth and experimental procedures. In a clean bench, an inoculation loop was used to pick up the revived OP50 culture and inoculate it onto the surface of LB solid medium using the streak plate method. The medium was inverted as well as incubated overnight at 37 °C to obtain single colonies. Afterward, the medium was placed in a 4 °C refrigerator for later use. Single colonies were picked and transferred to LB liquid medium as well as incubated overnight on a shaker at 37 °C to obtain OP50 culture. The culture was evenly spread onto NGM medium, avoiding the edges to prevent *C. elegans* from crawling onto the plate walls and drying out. After the plates were dried, they were incubated at 20 °C for 3 days before being used for *C. elegans* culture. Generally, 50 μL of bacterial suspension is spread on a 35 mm culture plate, 100 μL on a 60 mm culture plate, and 200 μL on a 90 mm culture plate.

#### 2.13.2. *C. elegans* Recovery

After removal from the −80 °C freezer, cryovials were thawed quickly by hand and the supernatant was discarded in a clean bench. Then, ~1 mL of the remaining *C. elegans* suspension was incorporated to the edge of the culture plate to prevent contamination of new bacterial growth by residual bacteria in the suspension.

After complete drying, the plate was placed in a 20 °C incubator. The recovery process was observed daily to monitor the recovery progress. When *C. elegans* reached a certain density, they were transferred to culture plates for routine cultivation. The recovered *C. elegans* were passaged 2–3 times before being used for subsequent experiments.

#### 2.13.3. *C. elegans* Culture and Passage

To minimize differences between samples, *C. elegans* need to be synchronized to obtain a large number of experimental samples. Alkaline hypochlorite treatment is most commonly used. Adult *C. elegans* containing eggs, cultured for ~4 days, are rinsed into sterile EP tubes with 3.5 mL of M9 buffer solution. Briefly, 1.5 mL of lysis buffer is added, and the tubes are vortexed for 6 min. After lysis, the tubes are centrifuged at 2500 rpm for 1 min, and the supernatant is removed. Under aseptic conditions, M9 was added to EP tubes for washing to remove lysis buffer. After shaking, the tubes were centrifuged and the supernatant was discarded. The washing process was repeated four times. The washed eggs were then mixed with a pipette and transferred to unsold areas of NGM agar. After ~12 h, the eggs had predominantly developed into L1-stage larvae, thereby completing the synchronization process.

During this process, the larvae actively crawled into the newly laid OP50 agar substrate, removing any remaining contaminants. The next day, the exposed agar surface could be removed with a scalpel. Careful control of the lysis time is crucial; too short a time will not allow the larvae to fully rupture, affecting the number of eggs; too long a time will damage the eggs and affect hatching.

Age-synchronized L4-stage wild-type N2 *C. elegans* with normal locomotor activity and no observable morphological defects were included in all experiments. Animals that crawled to the plate walls and desiccated, exhibited abnormal development, or were lost during transfer/washing steps were excluded from the analysis. These criteria were established a priori prior to the initiation of all in vivo experiments. For each assay, the exact number of animals included in the final analysis is reported in the corresponding results text and figure legends; no animals were excluded outside of the pre-specified criteria.

#### 2.13.4. *C. elegans* Culture

Synchronized L1 larvae were randomly allocated to each experimental or control group using a computer-generated random number sequence, with equal numbers of larvae transferred to each NGM plate within an experiment. To minimize confounding effects from incubator microenvironment variation, the position of all treatment plates within the 20 °C incubator was re-randomized daily throughout the culture period.

During perfusion culture, 2 g of aseptically prepared oleogel sample (with the same AST concentration or oleogel concentration used as a control group) was added to 200 mL of NGM agar medium. Simultaneously, bacterial suspensions of the same concentration were prepared and plated for subsequent use. Synchronized *C. elegans* larvae were transferred to the prepared plates as well as incubated at 20 °C. All culture procedures were performed under aseptic conditions, and age-synchronized *C. elegans* (L4 stage) were used in each experiment to ensure consistency in biological replication.

Sample size for all in vivo assays was determined a priori based on previously published *C. elegans* antioxidant and stress resistance assays, which demonstrated that 30 animals per group across 3 independent biological replicates provide a statistical power of >0.8 (1−β) to detect biologically meaningful differences in the primary outcome at a two-sided α level of 0.05. A total of 270 nematodes were included in the final analysis across all in vivo experiments (90 animals per treatment group, 30 per independent replicate).

#### 2.13.5. Determination of Lipofuscin Levels in *C. elegans*

On day 3, N2 *C. elegans* culture plates were exposed to heat shock at 35 °C for 1 h. The worms were then washed off the NGM plates using M9 buffer and subsequently rinsed 3–6 times with M9 buffer prewarmed to 35 °C in a water bath until the worm suspension became clear. After washing, the supernatant was discarded. The worms were fixed by adding 500 µL of 4% paraformaldehyde for 10 min, followed by the addition of M9 buffer. The samples were centrifuged at 3000 rpm, the supernatant was removed, and this washing step was repeated thrice. An appropriate number of worms were placed on a 2% agarose gel pad on a glass slide. Subsequently, images were captured via a fluorescence microscope (Olympus Corporation, Tokyo, Japan) under the DAPI channel. The fluorescence intensity (FI) was analyzed via the ImageJ software (version 1.54g, National Institutes of Health, Bethesda, MD, USA).

#### 2.13.6. Acute Heat Stress Test in *C. elegans*

The acute heat stress test was conducted using wild-type N_2_ *C. elegans*. On day 3 of culture, *C. elegans* from each group were transferred to new NGM plates and sealed with sealing film. The worms were then subjected to acute heat stress treatment at 35 °C for 5 h, starting at 20 °C. After treatment, the number of surviving *C. elegans* was counted: a platinum wire was used to gently touch the head or tail; if there was no movement response, the *C. elegans* were considered dead. The worms that crawled to the plate wall and dried out were considered escaped and not included in the survival count. Each experiment contained 30 *C. elegans* and was independently repeated three times.

#### 2.13.7. Determination of Reactive Oxygen Species Content

N2 *C. elegans* cultured for 3 days were exposed to a 35 °C heat shock environment for 1 h. The *C. elegans* were then washed from the plates with M9 buffer, and the washings were repeated 3–6 times with M9 buffer prewarmed to 35 °C until the *C. elegans* suspension was clear. After the centrifugation and removal of the supernatant, 1 mL of 100 μmol/L DCFH-DA fluorescent probe was incorporated, and the sample was incubated in a horizontal shaker in the dark for 30 min. After incubation, FI was measured via a microplate reader with an excitation wavelength of 488 nm as well as an emission wavelength of 525 nm.

#### 2.13.8. Determination of MDA Content and Antioxidant Enzyme Activity

On day 3, N2 *C. elegans* were subjected to heat shock at 35 °C for 1 h, then collected and washed with M9 buffer. After centrifugation, the supernatant was discarded and residual liquid removed with filter paper. The wet weight of *C. elegans* was accurately measured using the differential weighing method, M9 buffer was incorporated at a weight-to-volume ratio of 1:100, and the sample was sonicated under ice-bath conditions. After the centrifugation of the lysate, the supernatant was collected and stored at 4 °C for later use. Subsequently, the MDA content, SOD activity, and GSH-Px activity in the supernatant were determined according to the methods described in the respective kit instructions.

#### 2.13.9. Blinding of Outcome Assessment

All outcome assessments (nematode survival counting, fluorescence intensity quantification for lipofuscin and ROS, and biochemical assay measurements for MDA, SOD and GSH-Px) were performed by investigators blinded to group allocation. All samples were assigned unique coded labels prior to assay execution, with treatment identities masked until the full dataset was collected and statistically analyzed. Primary outcome measure: Survival rate of *C. elegans* under acute heat stress (35 °C for 5 h), which was pre-specified as the primary endpoint for a priori sample size determination. Secondary outcome measures: Lipofuscin accumulation, intracellular ROS levels, MDA content (a marker of lipid peroxidation), and endogenous antioxidant enzyme (SOD, GSH-Px) activities in nematodes post-heat stress.

### 2.14. Statistical Analyses

Data were expressed as mean ± standard deviation (SD) of three independent biological replicates (*n* = 3). One-way analysis of variance (one-way ANOVA) was used to evaluate significant differences among groups, with η^2^ reported as the effect size. Post hoc multiple comparisons were carried out using Tukey’s HSD test. A significance level was set at *p* < 0.05. Statistical analyses were performed using SPSS 26.0 (IBM Corp., Armonk, NY, USA) and graphs were plotted using GraphPad Prism 9.0 (GraphPad Software, San Diego, CA, USA).

## 3. Results and Discussion

### 3.1. Particle Size and Zeta Potential

**Figure 1:**
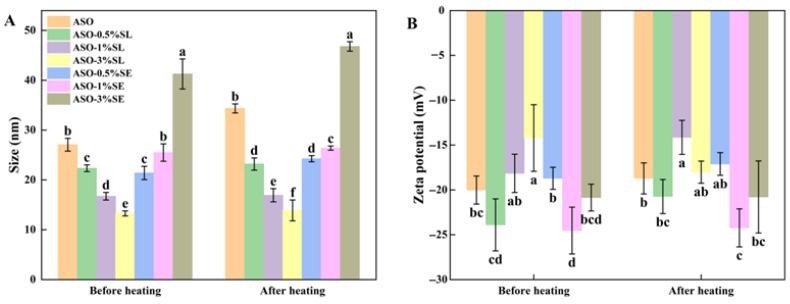
Particle size (**A**) and zeta potential (**B**) of different composite oleogel emulsions before and after heat treatment. Different letters in the same index indicate significant differences at *p* < 0.05.

As shown in Figure 1, all emulsions exhibited a significant increase in particle size and a significant decrease in zeta potential after boiling water bath treatment, consistent with the previously reported mechanism that high-temperature treatment disrupts the interfacial protein–polysaccharide layer, weakens steric hindrance and electrostatic repulsion, and promotes droplet flocculation or coalescence [32,33]. However, the magnitude of particle size increase differed significantly among systems, reflecting differences in thermal resistance. The pure SAE system showed the largest relative increase in particle size (26.9%, *p* < 0.05), indicating that the interfacial layer formed solely by the SP-AX complex had poor thermal stability. In contrast, emulsions supplemented with 1% SL or 1% SE showed the smallest particle size change after heating (*p* < 0.05 vs. pure SAE), confirming that 1% emulsifier concentration effectively improved interfacial compactness and thermal resistance. Notably, the 3% SE treatment group had significantly larger initial particle size and more severe aggregation after heating compared with the 1% SE group (*p* < 0.05), which may be attributed to interfacial overcrowding or competitive adsorption of excess emulsifier molecules. These results collectively demonstrate that emulsifier type and concentration are key factors regulating the interfacial structure and charge properties of protein–polysaccharide emulsions, which in turn determine the thermal stability of the system.

### 3.2. Microstructure of Oleogels

**Figure 2:**
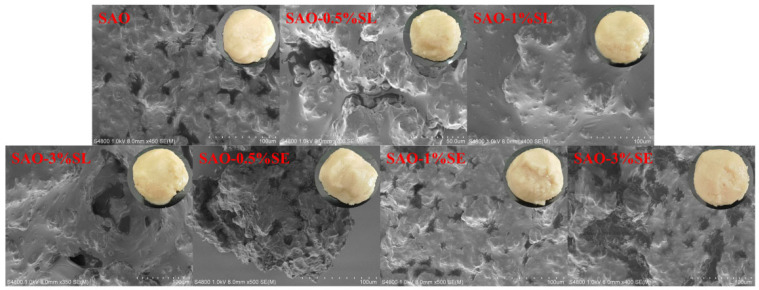
Macroscopic appearance and microstructure of different composite oleogels (magnification—1000).

As displayed in Figure 2, all samples of oleogels suggested a well-defined porous network structure after the oil removal process, and this result confirmed that the oil phase was well-encapsulated within a continuous three-dimensional network framework in the initial state [34]. Specifically, the basic matrix (soybean protein–arabinoxylan composite oleogels [SAO]) built from SA by glycosylation already had a relatively fine and dense pore structure. Adding 1% SL or SE further optimized the microstructure of the oleogel, resulting in a more uniform and continuous network arrangement, indicating that an appropriate amount of emulsifier effectively promoted the more stable assembly of the interfaces and crosslinking of the matrix. However, in case of increasing the emulsifier concentration up to 3%, the samples showed a significant increase in the pore size with structural roughening. This is probably attributed to interfacial overloading due to excessive emulsifier resulting in oil droplet flocculation or aggregation which in turn disrupts the network integrity [35]. This structural change trend confirms the particle size increase as well as zeta potential results measured by a laser particle size analyzer, together confirming the important effect of emulsifier concentration on the final microstructure as well as oleogel stability.

**Figure 3:**
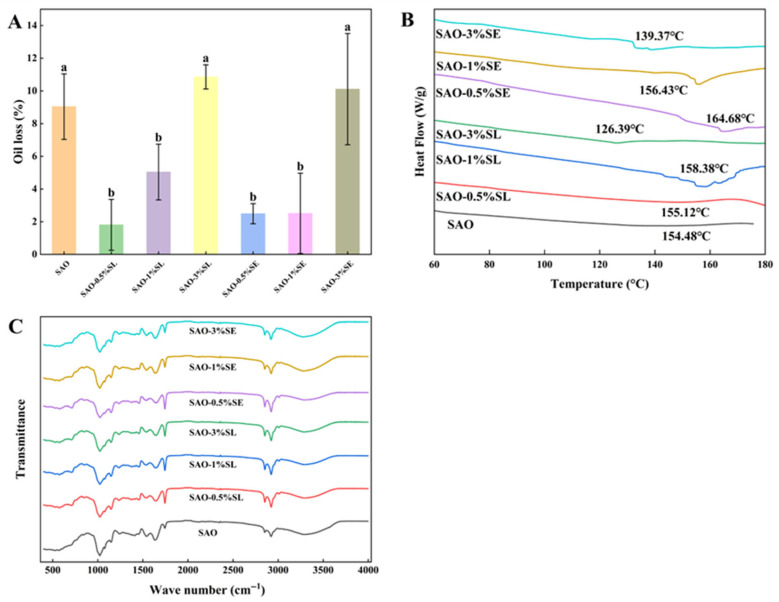
Oil loss rate (**A**), thermogram (**B**), and FTIR spectrum (**C**) of different composite oleogels. Different letters in the same index indicate significant differences at *p* < 0.05.

The oil loss rate of each oleogel sample is shown in Figure 3A. The blank SAO group had an oil loss rate of 9.04%, indicating that the protein–polysaccharide complex formed by glycosylation can construct a carrier matrix with certain stability. This is consistent with existing studies, where the molecular interaction between proteins and polysaccharides contributes to the formation of a structurally stable oleogel system; oil droplets are effectively encapsulated in the three-dimensional polymer network, and this structure has sufficient mechanical strength to resist external force damage during freeze-drying and subsequent processing [36]. The addition of low concentrations of emulsifiers (0.5% and 1%) significantly reduced the oil loss rate of oleogels (*p* < 0.05 vs. blank SAO). Specifically, the oil loss rates of SAO–0.5%SL, SAO–1%SL, SAO–0.5%SE and SAO–1%SE were decreased to 1.81%, 5.04%, 2.49% and 2.51%, respectively. This improvement is attributed to the orderly arrangement of an appropriate amount of SL or SE at the oil–water interface, which helps form a more continuous and stable three-dimensional network structure between SA molecules, thereby effectively inhibiting oil exudation. This finding is consistent with a previous study, where ester emulsifiers significantly improved the oil holding capacity of soybean protein oleogels, with the oil retention rate reaching 96.6% [18]. However, when the emulsifier concentration was increased to 3%, the oil loss rates of SAO–3%SL and SAO–3%SE were significantly increased to 10.86% and 10.11%, respectively (*p* < 0.05 vs. 1% treatment group and blank SAO), even higher than that of the emulsifier-free SAO group. This phenomenon is explained by emulsifier supersaturation, which leads to the self-aggregation of emulsifier molecules in the system or competitive interference with the original protein–polysaccharide network, further causing changes in interfacial properties and disruption of the gel network structure, which is also confirmed by the more porous and uneven structure in the SEM images.

### 3.3. Thermal Properties of Oleogels

Differential scanning calorimetry (DSC) was used to analyze the thermal properties of different soybean protein-based oleogels. The obtained heat flow curves (Figure 3B) directly reflect the energy changes associated with protein denaturation and network disruption, with the denaturation temperature (Tp) indicating structural stability and the enthalpy change (ΔH) quantifying the energy required for conformational unfolding and network disassembly [37]. The blank SAO system exhibited a Tp of 154.48 °C with a ΔH of 1.889 J/g, indicating the inherent thermal stability of the glycosylated SA matrix. The relatively low ΔH suggests a moderately ordered network structure. One-way ANOVA revealed a significant main effect of emulsifier treatment on Tp (F(6, 14) = 58.29, *p* < 0.001, η^2^ = 0.96) and ΔH (F(6, 14) = 37.62, *p* < 0.001, η^2^ = 0.94). At appropriate concentrations (0.5% and 1%), both SE and SL significantly increased the Tp of the system (*p* < 0.05). Specifically, the addition of 0.5% SE increased the Tp to 164.68 °C, which was 10.20 °C higher than that of the blank group. Concomitantly, the ΔH values increased markedly, reaching 2.989 J/g for SAO-0.5%SE and 7.017 J/g for SAO-1%SE. For SL-modified systems, ΔH increased from 1.709 J/g (0.5% SL) to 8.486 J/g (1% SL). The significant elevation in both Tp and ΔH indicates that an appropriate amount of emulsifier enhances the structural stability of the protein network through strengthened intermolecular interactions (e.g., hydrogen bonding), requiring more energy to disrupt the ordered gel structure [18]. However, when the emulsifier concentration was increased to 3%, the thermal stability of the system was significantly compromised. The denaturation peak temperatures of the 3% SE- and 3% SL-modified systems decreased to 139.37 °C and 126.39 °C, respectively, both significantly lower than that of the blank SAO group (*p* < 0.05). Correspondingly, ΔH values dropped sharply to 1.331 J/g for SAO-3%SE and 0.2824 J/g for SAO-3%SL. This substantial reduction in both Tp and ΔH suggests that excess emulsifier molecules interfere with the ordered assembly of the protein–polysaccharide network, likely via competitive adsorption and disruption of key intermolecular interactions, resulting in a less stable, more disordered gel structure [19]. These findings collectively demonstrate that emulsifier type and concentration act in tandem to govern both the direction and magnitude of their effects on the thermal stability and structural order of protein-based oleogel systems.

### 3.4. FTIR Analysis of Oleogels

FTIR spectroscopy is an extensively leveraged analytical method for characterizing functional groups and intermolecular interactions in substances [38]. Through comparative analysis (Figure 3C), no pronounced new peaks or remarkable peak shifts appeared in the FTIR spectra of all samples, suggesting the addition of emulsifier did not form new chemical bonds with gel molecules, and the interaction was mainly physical bonding. Each oleogel sample showed several common absorption peaks in the characteristic wavenumber region. Among these peaks, the peak at 1743 cm^−1^ is the stretching vibration peak of –C=O in the ester bond; the peaks at 2851 as well as 2921 cm^−1^ are because of the symmetric and asymmetric stretching vibrations of –CH_2_, while the absorption at ~3009 cm^−1^ corresponds to the stretching vibration of C–H in the cis double bond. This indicates that the oil contains a high content of linoleic acid [39]. In addition, characteristic peaks of amide I as well as amide II were seen at 1642 and 1536 cm^−1^. The absorption peak at ~1234 cm^−1^ was due to C–N stretching as well as N–H bending vibrations in amide III [40], where amide I is often used for analyzing protein secondary structure [41], and the research results align with prior reports. Notably, the broad and strong absorption band in 3100–3700 cm^−1^ is due to the O–H stretching vibration, which may be related to the formation of intermolecular or intramolecular hydrogen bonds [15]. Hydrogen bonds are one of the crucial forces for maintaining the 3D network structure stability of oleogels [42]. Experiments have shown that the intensity of the O–H stretching vibration peak changes significantly with the addition of emulsifier, indicating that the hydrogen bond interaction is enhanced [43]. Therefore, the introduction of an appropriate amount of emulsifier can further improve the overall stability of the oleogel structure by enhancing non-covalent interactions such as hydrogen bonds.

### 3.5. Rheological Behavior

**Figure 4:**
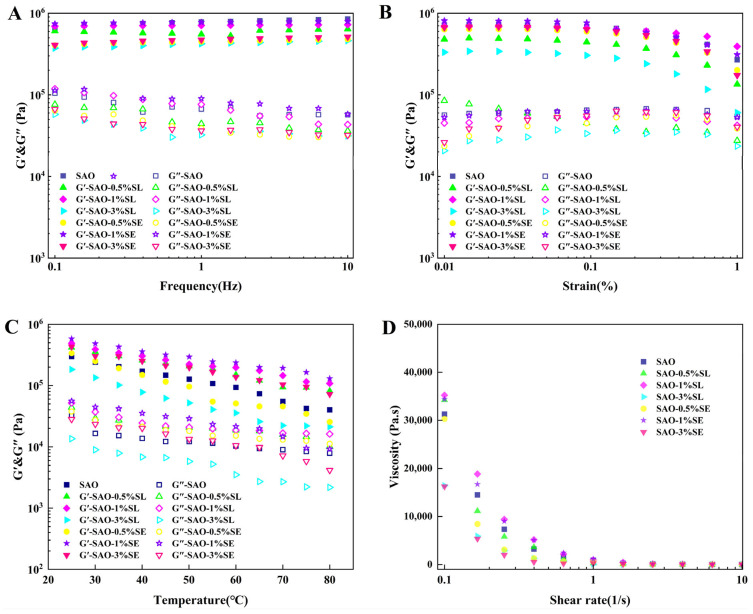
Rheological data of various oleogels: (**A**) frequency scan (0.1–10 Hz); (**B**) strain scan (0.01–1%); (**C**) temperature scan (20–80 °C); (**D**) viscosity measurement (0.1–10 s^−1^).

The oleogels’ rheological properties are important performance attributes that directly influence their texture, stability, and application in food systems. In the frequency sweep (0.1–10 Hz) (Figure 4A), the storage modulus (G′) was consistently significantly greater than the loss modulus (G″) and G′ increased with increasing frequency with all samples displaying typical solid-like elastic behavior [44]. The higher G′ value indicates a higher three-dimensional network structure and better mechanical strength [45]. Results suggested adding 1% SL or SE had no remarkable effect on the G′ of oleogel, which led to the conclusion that at the concentration, the emulsifier did not weaken the basic strength of the gel network, and the system had high mechanical properties. Furthermore, G′ showed low dependence on frequency changes in the whole frequency range, suggesting the prepared oleogel had good mechanical stability. The result is in agreement with the rheological behavior of oleogel systems built on the basis of the emulsion template method in previous studies [46,47]. The resistance of the samples to permanent deformation was tested by strain scanning tests (Figure 4B) [19]. Strain scanning results show that all oleogels had rheological properties dominated by elasticity with G″ higher than G″ throughout the linear viscoelastic region (LVR) [48]. The strain range of the LVR for all samples was 0.01–0.1%; when the strain value was higher than the critical value of 0.1%, G′ and G″ began to decrease, indicating that larger strains cause the destruction of the 3D network structure within the oleogel, which resulted in the loss of its semi-solid properties. This trend aligns with the previously reported studies [19]. Yet, when the emulsifier concentration rose to 3%, the G′ of all samples decreased remarkably. This change can be because of the changes in microstructure; the high concentration of emulsifier interfered with the ordered assembly of the protein–polysaccharide matrix, causing the formation of a discontinuous network with larger pores as well as poor uniformity and thus weakening the overall gel structure.

The temperature response characteristics of oleogel structures were evaluated using temperature sweep tests (Figure 4C). With increasing temperature, the G′ as well as G″ values of all samples suggested a gradual decrease, suggesting elevated temperature weakens non-covalent interactions such as hydrogen bonds within the gel network, thereby leading to structural softening [49]. The phenomenon aligns with the results seen in zein oleogels, further confirming that temperature has a universal effect on the stability of protein-based gel networks [46]. Notably, the samples supplemented with 1% emulsifier showed higher viscoelastic moduli, and the modulus variation was more stable during heating. This may be attributed to the appropriate amount of emulsifier promoting effective binding between protein and polysaccharide molecules, forming a denser and more stable three-dimensional network structure. Among all tested samples, G′ remained higher than G″ throughout the entire temperature range of 20 °C to 80 °C, demonstrating that the oleogels maintained their inherent solid-like viscoelastic characteristics without undergoing gel–sol transition, thus exhibiting excellent resistance to external temperature changes [50]. As shown in Figure 4D, adding emulsifier significantly affected the apparent oleogel viscosity, with the sample containing 1% emulsifier exhibiting the highest initial viscosity. All oleogel systems exhibit typical shear-thinning characteristics, meaning that apparent viscosity gradually decreases with rising shear rate. This rheological behavior is mainly because of the reversible disruption of the 3D network structure within the oleogel under shear stress [51].

### 3.6. Encapsulation Efficiency (EE) of AST in Oleogels

**Figure 5:**
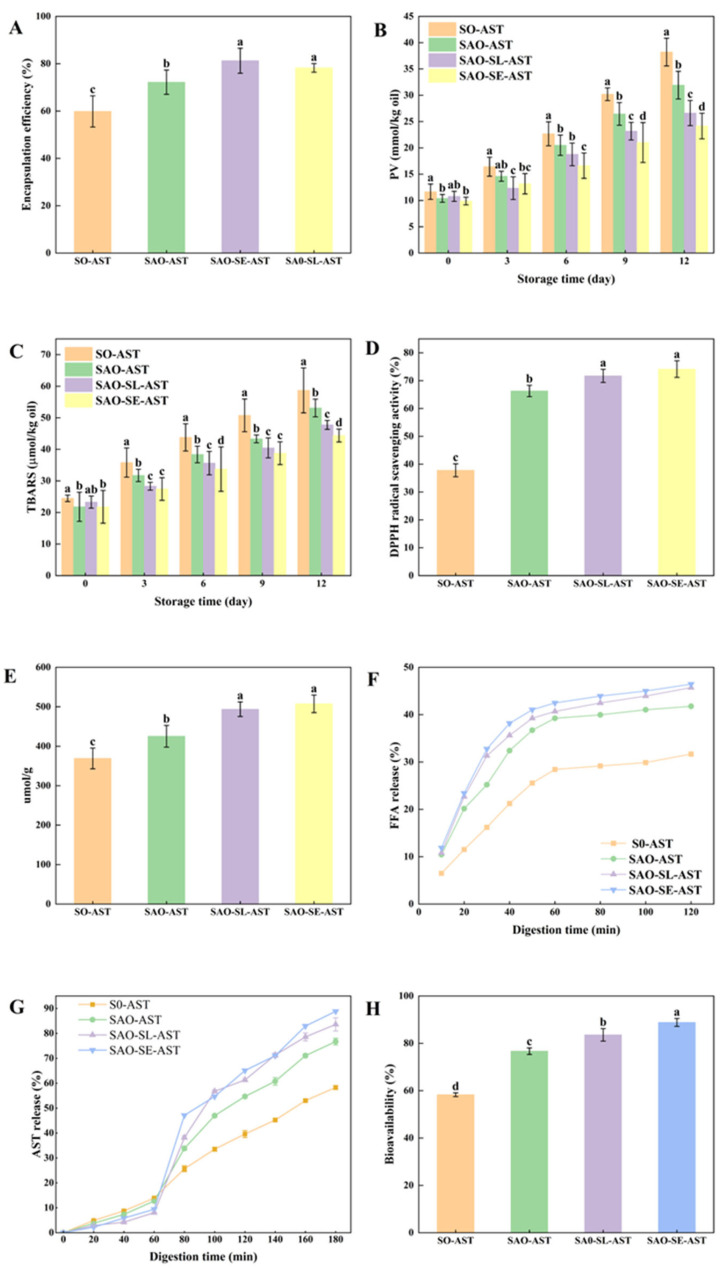
AST encapsulation efficiency of different composite oleogels (**A**); changes in peroxide value (**B**) and thiobarbituric acid reactants (**C**) during 12 days of storage; DPPH free radical scavenging activity (**D**) and total antioxidant capacity (**E**); FFA release (**F**) and AST release (**G**) during in vitro simulated digestion; and AST bioavailability (**H**). Vertical lines represent standard deviations (SD, *n* = 3). Different letters within the same index indicate significant differences at the *p* < 0.05 level.

The Figure 5A shows significant differences in the encapsulation efficiency (EE) of astaxanthin (AST) among different oleogel samples (F(3, 8) = 15.72, *p* < 0.001, η^2^ = 0.85), specifically SAO-PC-AST (78.24 ± 1.82%), SAO-SE-AST (81.27 ± 5.27%), SO-AST (59.85 ± 6.59%), and SAO-AST (72.22 ± 5.12%). SO-AST exhibited significantly lower EE, while the introduction of arabinoxylan via glycosylation significantly improved the EE of SAO-AST. This was attributed to the formation of a soy protein–arabinoxylan conjugate, which improved emulsification by increasing steric stability, enhancing interfacial adsorption, and forming a more cohesive network around oil droplets during homogenization [52,53]. Similarly, studies have suggested that adding 0.5% and 1.0% of seaweed sulfated polysaccharides to gelatin oleogels can significantly improve the EE of AST [49]. The incorporation of SL and SE once again significantly improved the EE of AST, which may be attributed to their enhancement of the structural density within the composite network. The stable network that formed enhanced the “physical barrier” effect on the oil phase and AST.

### 3.7. Oxidation Stability and Antioxidant Capacity

Oxidative stability and antioxidant capacity are key factors affecting the flavor and nutritional quality of oleogel products. During storage, lipid oxidation will lead to flavor deterioration and nutritional value loss, which seriously affects the overall quality of the product [48]. To evaluate the oxidative stability of oleogels during storage, a 12-day accelerated oxidation test was carried out at 40 °C, and the changes in PV and TBARS value were measured every 3 days (Figure 5B,C). The results showed that the PV and TBARS values of all oleogels increased significantly with the extension of storage time, but the oxidation rate of different composite systems was significantly different (*p* < 0.05). Compared with SO–AST and SAO–AST, the composite oleogels with emulsifiers had significantly lower PV and TBARS values at the same storage time point (*p* < 0.05). The reduction in these oxidation indicators is attributed to the fact that the emulsifiers enhance the encapsulation effect of the gel matrix on oil droplets, making the oil phase tightly bound in the oleogel network structure, effectively blocking the contact between oil and oxygen, limiting the migration and diffusion of free radicals and pro-oxidants, and ultimately slowing down the lipid oxidation process [48]. For in vitro antioxidant capacity, DPPH free radical scavenging assay was first performed to evaluate the free radical quenching ability of different oleogels (Figure 5D). The DPPH radical scavenging rates of SO–AST, SAO–AST, SAO–SL–AST and SAO–SE–AST were 37.84%, 40.12%, 61.16% and 67.79%, respectively. SAO–AST showed a markedly higher scavenging rate than SO–AST (*p* < 0.05), and emulsifier incorporation further boosted the radical scavenging capacity of the oleogel systems (*p* < 0.05 vs. SAO–AST). The total antioxidant capacity, measured via FRAP assay, followed an identical trend to the DPPH radical scavenging results (Figure 5E). The total antioxidant capacity of SO–AST was 369.06 ± 26.30 μmol/g, which rose to 425.32 ± 27.56 μmol/g for SAO–AST (*p* < 0.05). Notably, emulsifier addition further enhanced this parameter, with values reaching 493.77 ± 18.41 μmol/g for SAO–SL–AST and 507.60 ± 22.24 μmol/g for SAO–SE–AST; significant differences were observed between all groups, as indicated by different superscript letters (*p* < 0.05). These results collectively confirm that AX glycosylation modification effectively improves the free radical scavenging ability and total antioxidant capacity of SPI-based oleogels, and the addition of an appropriate amount of food-grade emulsifiers further amplifies this antioxidant effect. This enhancement is driven by two key mechanisms. First, non-covalent interactions between the SA glycosylated complex and emulsifier molecules drive tighter protein adsorption at the oil–water interface, forming a denser, more continuous interfacial protective film. This physical barrier effectively blocks the diffusion of pro-oxidants and free radicals into the oil phase, reduces lipid peroxidation, and minimizes degradation of encapsulated AST, thereby preserving the intrinsic antioxidant activity of the bioactive [54,55]. Second, the higher AST encapsulation efficiency in emulsifier-modified oleogels ensures greater retention of active AST, which directly contributes to the stronger free radical scavenging and total antioxidant capacity of the system. These findings align with existing reports that the antioxidant efficacy of lipophilic carotenoids in delivery systems is closely tied to the structural stability of the carrier matrix and the retention rate of the active compound [11].

### 3.8. FFA and AST Release and AST Bioavailability During In Vitro Simulated Digestion of Oleogels

This study systematically evaluated the digestive characteristics of different oleogel samples by analyzing the release of FFAs. As shown in Figure 5F, during the 120 min in vitro digestion period, all oleogel samples showed a typical pattern of FFA release: an initial linear increase after a gradual leveling off in the later stage. This tendency suggests that prepared oleogel network structure has appropriate compactness, which can encapsulate the target oil, and form natural controlled release system for lipid digestion, preventing lipid hydrolysis. The type of gel had a remarkable effect on the amount of FFA release. At the end of digestion, the FFA release rate of SO was 31.68% but that of SAO increased significantly to 41.76%. The FFA release rate of SAO-SL and SAO-SE was further increased to 45.72% and 46.44%. The results show the complexation of polysaccharides greatly favors the lipolysis of oleogels, and adding emulsifiers increases even more the susceptibility of the oil to the hydrolysis of lipase. The underlying regulatory mechanisms can be explained by three main aspects. First, the presence of arabinoxylan changes the surface properties and molecular conformation of the oleogel, resulting in a more expanded state of the gel molecules. This expanded state makes it easier to expose the enzyme-binding sites, increasing the binding affinity of lipase to the oil as well as increasing the oil bioaccessibility [16]. Second, lipid hydrolysis is dependent on the formation of colloidal structures providing an extensive, accessible interface for enzymatic activity. The addition of emulsifiers helps to optimize the compatibility of the oil and water system, providing a favorable microenvironment for the catalysis of lipase [56]. Third, the synergistic effect of the polysaccharide and emulsifiers promotes the hydrogen bond formation in the gel network as well as greatly improving the water absorption and the swelling capacity of the gel. This increased absorption and swelling increase the penetration as well as activity of digestive enzymes into the gel matrix, which eventually increases the efficiency of lipid digestion [57,58].

The release kinetics of AST from different oleogels during simulated gastrointestinal digestion are shown in Figure 5G. AST exhibited a typical two-stage release characteristic of “low release in the gastric phase and high release in the intestinal phase”, and the gel matrix type had a significant effect on the release behavior of AST in each digestion stage (*p* < 0.05). In the simulated gastric digestion stage (pH 2.5, 1 h), the AST release rate of all oleogels was maintained at a low level. Among them, the SO group had an AST release rate of 15.90%, indicating that the single protein matrix was easily degraded by pepsin, and thus could only provide limited protection for AST. In contrast, the AST release rates of the SAO, SAO-SL and SAO-SE groups were significantly reduced to 8.05–11.72% (*p* < 0.05), confirming that the introduction of polysaccharide complexes and emulsifiers can significantly enhance the protective effect of the gel matrix on AST in the gastric phase. The underlying mechanism is that the steric hindrance of arabinoxylan molecules on the gel surface blocks the direct contact between pepsin and the gel matrix [59]; meanwhile, the addition of emulsifiers promotes the formation of a denser three-dimensional network structure, reduces the hydrophobicity of the composite oleogel, and may slightly inhibit the catalytic activity of pepsin, thereby reducing the premature leakage and degradation of AST in the acidic gastric environment [49]. When the digestive environment was switched from acidic gastric fluid to neutral intestinal fluid (pH increased from 2.5 to 7.0), all oleogel samples showed a rapid release of AST. At the end of intestinal digestion, the AST release rate of the SAO group was 76.69%, while those of the SAO-SL and SAO-SE groups were further significantly increased to 83.58% and 88.84%, respectively (*p* < 0.05 vs. SAO), all of which were significantly higher than that of the SO group (58.29%, *p* < 0.05). The significant change in pH is the key trigger for the large-scale release of AST: the neutral environment triggers the reconstruction of the structure and solubility of the gel matrix, and the gradual degradation of the gel network, thereby efficiently releasing the encapsulated AST. In contrast, the incorporation of emulsifiers optimizes the pH responsiveness and network stability of the gel, reduces the degradation of the matrix by gastric enzymes, and achieves more sufficient AST release in the intestinal environment, showing excellent targeted delivery performance of active ingredients [49].

Bioaccessibility, which is defined as the percentage of bioactive compounds transferred from the lipid phase into the aqueous mixed micellar phase after in vitro oral, gastric, and small intestinal digestion, is an important indicator for predicting the intestinal absorption efficiency [11]. For AST to be released for uptake by intestinal epithelial cells, it must first be released from the oleogel matrix for it to be incorporated into the micellar phase. Results show the type of gel has an impressive impact on the bioaccessibility of AST (Figure 5H). Compared with SO, the AST bioaccessibility of SAO was significantly improved, and SAO-SL and SAO-SE showed further improvements. These values showed a strong positive correlation with the intestinal phase AST release rate, indicating that the complexation with polysaccharides and the incorporation of emulsifiers create favorable conditions for the micellization of AST by modifying the digestive behavior of the oleogels. Further analysis confirmed a positive connection of AST bioaccessibility with the amount of FFA release. This trend is consistent with existing findings in carotenoid micellization studies, i.e., the degree of carotenoid micellization is similarly correlated with the release behavior of FFAs during digestion [60]. These consistencies suggest that FFAs produced during lipolysis promote the incorporation of bioactive compounds in mixed micelles, thus increasing their bioaccessibility. In addition, polysaccharides and emulsifiers raise steric hindrance and system viscosity in the digestive environment through their interactions with the gel matrix [61]. This helps to improve the chemical stability of AST and reduces its degradation during the course of digestion. Such a protective effect is consistent with the previous reports of a polysaccharide–protein composite carrier being able to form physical barriers to minimize bioactive degradation, which highlights the advantage of composite oleogel systems for the protection as well as delivery of lipophilic active components.

While the static INFOGEST model provides reproducible insights into bioaccessibility, it lacks the dynamic mechanical forces (peristalsis) and mucosal absorption present in vivo. Furthermore, the absence of an oral phase (mastication, salivary amylase) may underestimate the initial structural breakdown of oleogels. These limitations should be considered when extrapolating to human physiological conditions.

### 3.9. In Vivo Antioxidant Analysis of AST-Loaded Oleogels

**Figure 6:**
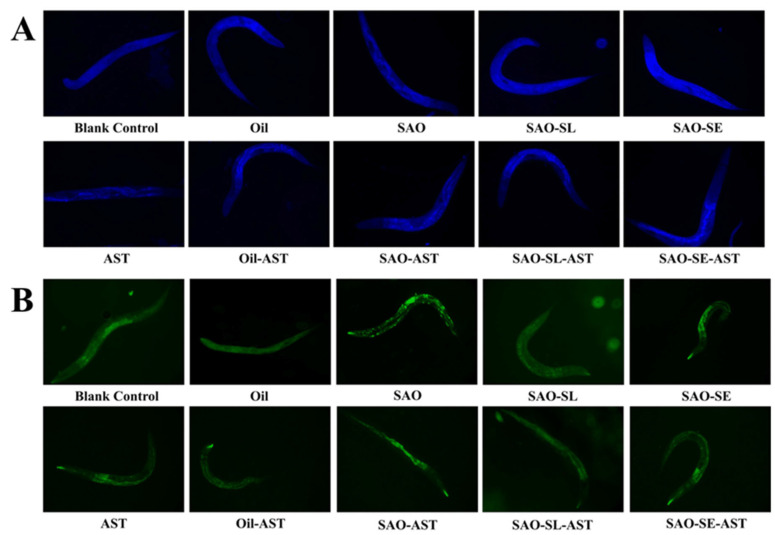
Fluorescence microscopy analysis of lipofuscin accumulation and reactive oxygen species (ROS) levels in *C. elegans* under heat stress with different culture conditions. (**A**) Lipofuscin autofluorescence imaging, where the blue fluorescence signal represents lipofuscin accumulation in nematodes; (**B**) ROS fluorescence imaging, where the green fluorescence signal represents the intracellular ROS level in nematodes.

**Figure 7:**
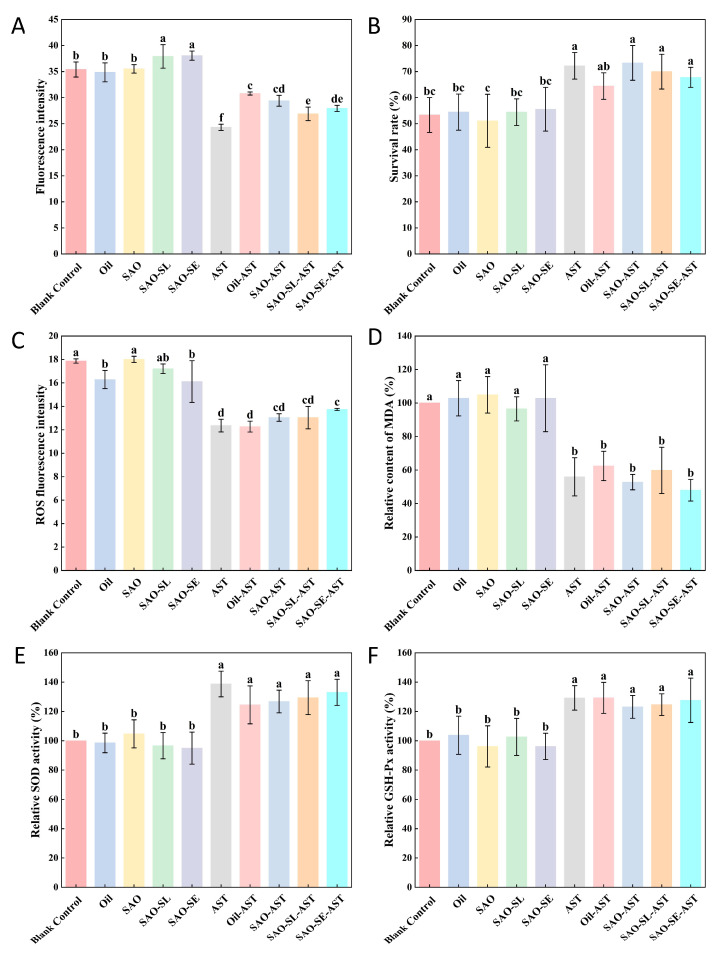
Lipofuscin fluorescence intensity (**A**); survival rate (**B**); ROS fluorescence intensity (**C**); relative MDA content (**D**); relative SOD activity (**E**); relative GSH-Px activity (**F**). Different letters within the same index indicate significant differences at the *p* < 0.05 level.

Following heat stress exposure, fluorescence imaging revealed distinct patterns of lipofuscin and ROS accumulation in *C. elegans* across treatment groups (Figure 6A,B). Quantification of lipofuscin fluorescence intensity confirmed significant intergroup differences (Figure 7A). While free AST significantly suppressed lipofuscin deposition (28.3% reduction vs. blank control, *p* < 0.01), oleogel formulations further enhanced this inhibitory effect: SAO-SE-AST treatment reduced lipofuscin accumulation by 41.7% (*p* < 0.01) relative to the blank control, outperforming the free AST group (*p* < 0.05). Matrices devoid of AST showed no significant inhibitory effect on lipofuscin deposition, confirming that the observed effects were driven by AST delivered via the oleogel system.

Acute heat stress was used to induce endogenous oxidative stress, with survival rate under thermal challenge as the pre-specified primary outcome [62]. Post-heat stress treatment, one-way ANOVA revealed a significant main effect of treatment on nematode survival rate (F(9, 20) = 10.274, *p* < 0.001, η^2^ = 0.822). Survival rates in all AST-loaded oleogel groups ranged from 64.44% to 73.33%, significantly higher than the blank control (28.89%, *p* < 0.01) and comparable to the free AST positive control (62.22%, Figure 7B). This result demonstrates that oleogel-based delivery markedly enhances organismal thermotolerance in *C. elegans*. Intracellular ROS levels, measured immediately post-heat stress, were 3.2-fold higher in the blank control group relative to the non-stressed baseline (Figure 7C). All AST-containing treatments effectively suppressed ROS overproduction, with the SAO-SE-AST group reducing ROS levels to 42.6% of the blank control (*p* < 0.01). This finding indicates that AST mitigates heat-induced oxidative damage primarily by curbing excessive ROS generation, with the enhanced effect of the oleogel formulation linked to improved AST bioavailability.

As a key marker of lipid peroxidation, MDA content reflects the severity of oxidative cellular injury [63]. AST-loaded oleogels reduced MDA levels in heat-stressed nematodes to 47.94–57.79% of the blank control (Figure 7D), an effect comparable to or stronger than free AST, confirming that the oleogel delivery system attenuates heat-triggered lipid peroxidation and associated oxidative injury. Enzymatic analysis further revealed that AST-loaded oleogels significantly augmented the activities of key endogenous antioxidant enzymes: SOD activity increased by 26.77–33.04%, and glutathione peroxidase (GSH-Px) activity increased by 23.13–27.61% relative to the blank control (Figure 7E,F). These results imply that AST delivered via the oleogel system activates the endogenous antioxidant defense system, enhancing enzymatic ROS scavenging capacity to counteract stress-induced oxidative damage [64]. Notably, the superior in vivo performance of the SAO–SE–AST (1%) group aligns coherently with the established “microstructure–digestion–bioactivity” relationship. The optimally structured network achieved with 1% SE—characterized by enhanced interfacial compactness and gel density—directly enabled the highest AST encapsulation efficiency (81.27%) and bioaccessibility (88.84%). This efficient delivery ensured greater systemic availability of bioactive AST, which subsequently translated to the most pronounced reductions in oxidative biomarkers (lipofuscin, ROS, MDA) and the strongest upregulation of antioxidant enzymes. Thus, the cascade from rational microstructural design, through controlled gastrointestinal transit and release, to potent in vivo antioxidant efficacy, validates microstructure engineering as a decisive strategy for optimizing lipophilic nutraceutical delivery. In summary, AST-loaded oleogels effectively lower ROS and MDA levels, boost endogenous antioxidant defenses, and significantly improve survival in heat-stressed *C. elegans*, with efficacy directly governed by the engineered oleogel architecture.

## 4. Conclusions

In this work, we fabricated AST-loaded oleogels using a soy protein–arabinoxylan (SA) glycosylation complex as a food-grade gelator, paired with two widely used food-grade emulsifiers, sucrose ester (SE) and soy lecithin. We found that both the type and concentration of emulsifier acted as critical regulators of the system’s interfacial architecture and three-dimensional network compactness. The optimal performance was achieved at 1% emulsifier addition, which simultaneously improved the thermal stability, rheological strength, and AST encapsulation efficiency (81.27%) of the oleogels. Furthermore, these optimized formulations showed markedly improved oxidative stability, with lower peroxide value (PV) and thiobarbituric acid reactive substance (TBARS) values throughout accelerated storage, along with stronger in vitro antioxidant activity as measured by DPPH radical scavenging and FRAP assays. These findings confirm that the optimized oleogels can act as robust functional carriers to protect lipophilic bioactives from oxidative degradation during processing and storage. The optimized oleogel system also enabled gastric slow-release and intestinal-targeted delivery of AST, reaching a maximum bioaccessibility of 88.84% and significantly enhancing its in vitro bioavailability. In wild-type *C. elegans*, the AST-loaded oleogels effectively reduced lipofuscin accumulation, improved survival under acute heat stress, mitigated intracellular ROS overproduction, and upregulated the activity of endogenous antioxidant enzymes. Beyond their well-established role as emulsion stabilizers, food-grade emulsifiers can function as key architectural modulators in protein–polysaccharide oleogel systems. This work establishes a robust quantitative “microstructure–digestion–bioactivity” correlation, providing a practical, rational framework for the design of high-performance, label-friendly delivery systems for sensitive lipophilic nutraceuticals. Future work will focus on validating these findings in mammalian models and optimizing the formulation for scalable industrial production.

## Data Availability

The original results presented in this study are included in the article/Supplementary Materials. If you have further questions, please contact the corresponding authors.

## Supplementary Materials

The following supporting information can be downloaded at: https://www.mdpi.com/article/10.3390/foods15081315/s1, Figure S1: Influence of sucrose ester (SE) and sucrose laurate (SL) concentration on the oil binding capacity of oleogels (pre-experiment screening).
