# Supplementary material for: Emulsifier-Modulated Microstructure of Soy Protein–Arabinoxylan Oleogels Improves Astaxanthin Bioaccessibility and In Vivo Antioxidant Activity
Source: Foods. 2026 Apr 10;15(8):1315. doi: 10.3390/foods15081315 (PMC13114323; doi:10.3390/foods15081315)
Supplement: Supplementary file 1 [file foods-15-01315-s001.zip › foods-4223010-supplementary.pdf]

**Supplementary S1**

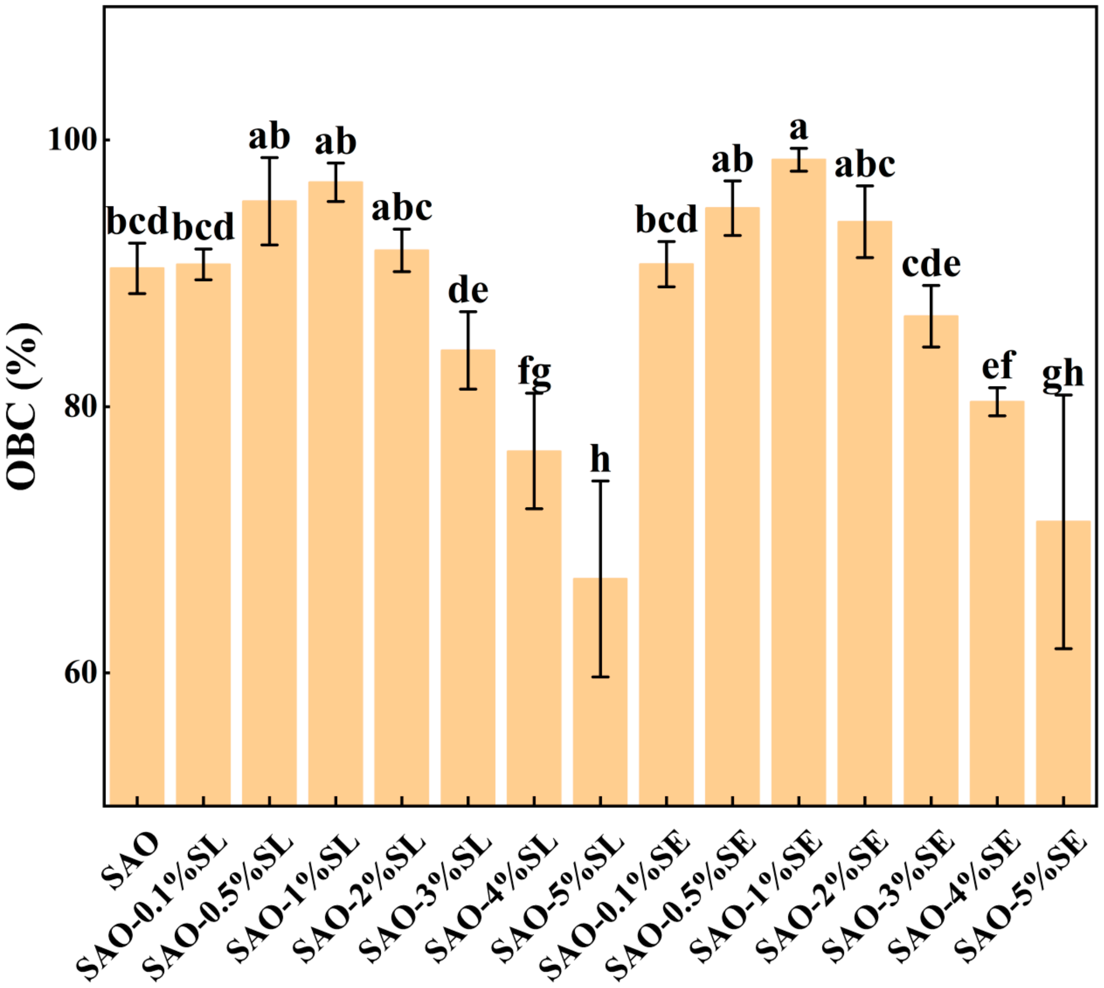


Influence of sucrose ester (SE) and sucrose laurate (SL) concentration on the oil binding capacity of oleogels (pre-experiment screening)
